# Chemical composition and biological activities of essential oils from six lamiaceae folk medicinal plants

**DOI:** 10.3389/fpls.2022.919294

**Published:** 2022-08-01

**Authors:** Jiahui Sun, Peipei Sun, Chuanzhi Kang, Lanyue Zhang, Lanping Guo, Yaping Kou

**Affiliations:** ^1^National Resources Center for Chinese Materia Medica, China Academy of Chinese Medical Sciences, Beijing, China; ^2^Institute of Food Science and Technology, Chinese Academy of Agriculture Sciences, Beijing, China; ^3^Guangdong Provincial Key Laboratory of Plant Resources Biorefinery, School of Biomedical and Pharmaceutical Sciences, Guangdong University of Technology, Guangzhou, China; ^4^National Center of China for Flowers Improvement, Institute of Vegetables and Flowers, Chinese Academy of Agricultural Sciences, Beijing, China; ^5^Key Laboratory of Biology and Genetic Improvement of Flower Crops (North China), Ministry of Agriculture and Rural Affairs, Beijing, China

**Keywords:** Lamiaceae herb, essential oil, chemical composition, folk medicinal plants, biological activities

## Abstract

Essential oils have attracted wide attention in recent years due to their extensive applications in natural functional ingredients, pharmaceutical preparations, biomedical products, and the cosmetics industry. In this study, the chemical compositions and biological activities of essential oils extracted from six Lamiaceae herbs, including *Pogostemon cablin* (Blanco) Benth. (PCEO), *Perilla frutescens* (L.) Britton (PFEO), *Salvia japonica* Thunb. (SJEO), *Rosmarinus officinalis* L. (ROEO), *Lavandula angustifolia* Mill. (LAEO), and *Agastache rugosa* (Fisch. & C. A. Mey.) Kuntze (AREO), were determined and analyzed. A total of 167 components were identified from the six essential oils by GC-MS analysis, with 35, 24, 47, 46, 54, and 37 components in PCEO, PFEO, SJEO, ROEO, LAEO, and AREO, respectively. Hierarchical cluster analysis of chemical compositions showed that the composition of the six essential oils was significantly different in content, and they were clearly divided into six classes. However, all of these six essential oils exhibited promising anti-inflammatory activity by inhibiting the expression of interleukin-1, interleukin-6, tumor necrosis factor-α, and cyclooxygenase-2 in rats with adjuvant arthritis, among which PFEO had the best performance. In addition, the six essential oils displayed significant cytotoxicity on B16 (IC_50_ = 86.91–228.91 μg/mL) and LNCaP cell lines (IC_50_ = 116.4–189.63 μg/mL). Meanwhile, all of them presented satisfactory antioxidant activity (IC_50_ = 4.88–13.89 μg/mL) compared with Trolox C (IC_50_ = 13.83 μg/mL), and SJEO (IC_50_ = 7.93 μg/mL) served as an optimal candidate natural antioxidant by DPPH assay. Taken together, these results indicate that the six Lamiaceae essential oils manifest excellent and diverse biological activities, enabling them to be used as perfect natural functional ingredients in antioxidant, antitumor, or anti-arthritic drugs. This study provides more references for pharmaphylogeny research and drug discovery from folk medicinal plants.

## Introduction

The Lamiaceae family contains 236 genera and about 7,173 species, almost cosmopolitan, except for the coldest regions of high latitude. Seven diversity centers were recognized: (1) Mediterranean and SW Central Asia; (2) Africa south of the Sahel and Madagascar; (3) China; (4) Australia; (5) South America; (6) Northern America and Mexico; (7) Indomalesian region (SE Asia). Based on their distribution, these species-rich genera fall generally into two groups: those mostly tropical in origin, including Vitex (Viticoideae), Clerodendrum (Ajugoideae), Callicarpa (Incertae sedis), Ocimum, Hyptis (Nepetoideae), and those which probably had a temperate origin, but often extend into the montane tropics, such as Ajuga, (Ajugoideae), Scutellaria (Scutellarioideae), Stachys (Lamioideae), Salvia, Clinopodium, Mentha, (Nepetoideae). In addition, Teucrium (Ajugoideae) has a distinct distribution in the southern hemisphere, but is most species-rich in the north, spreading down to the mountains of tropical Africa. The Lamiaceae plants contain many aromatic and medicinal plants that are widely used in traditional and modern medicine, food, and cosmetics industries (Vukovic et al., 2009; Nieto, 2017; Borges et al., 2019). In traditional and modern medicine, some Lamiaceae species, such as *Perilla frutescens* (L.) Britton, *Pogostemon cablin* (Blanco) Benth., *Rosmarinus officinalis* L., *Lavandula angustifolia* Mill., and *Agastache rugosa* (Fisch. & C. A. Mey.) Kuntze, are pervasively applied to dispel fever, expel superficial evils, eliminate stasis, induce diuresis, promote blood circulation, and reduce edema (Guo et al., 2016; Luo et al., 2019). *R. officinalis*. has been used as an analgesic, antispasmodic, and antidepressant to cure intercostal neuralgia, headaches, migraine, insomnia emotional upset, and depression in folk medicine (Ghasemzadeh Rahbardar and Hosseinzadeh, 2020). For a long time, the biological activities of extracts from these plant species have been studied, such as the antitumor, antioxidant, antimicrobial, and anti-inflammatory activities (Nieto, 2017; Guo et al., 2019; Karpinski, 2020).

Essential oil, an important category of plant extracts, has a multidirectional action mode and a variety of biological activities (Wojtunik-Kulesza et al., 2019). Essential oils can disrupt the cell and cell membrane via a permeabilization process. The lipophilic compounds of essential oils can pass through the cell wall; damage polysaccharides, fatty acids, and phospholipids; change the permeability for H^+^ and K^+^ cations to affect cellular pH; and damage organelles and disintegrate mitochondrial membrane (Karpinski, 2020). What is more, essential oils inhibit the biosynthesis of fungal DNA, RNA, proteins, and so on. They are widely applied in cosmetic additives, natural functional food, pharmaceutical preparations, and biomedical products (Nieto, 2017; Zhang et al., 2017b, 2020a). Specifically, essential oils from medicinal plants have attracted increasing attention in recent years for their multifaceted biological activities and diverse chemical compositions (Santos and Rao, 2000; El-Sayed et al., 2014; Xue, 2016). Lamiaceae family plants rich in essential oils have significant values in natural medicine, pharmacology, cosmetology, and aromatherapy. Some Lamiaceae species that are used in traditional medicine have been employed in the characterization of their essential oils, such as bioactivities and phytochemical composition. For example, essential oils of *P. cablin* (PCEO), *P. frutescens* (PFEO), *R. officinalis* (ROEO), and *L. angustifolia* (LAEO) are mainly composed of patchouli alcohol, linalool, α-terpineol, β-pinene, _DL_-menthol, and isobornyl acetate, which show strong anti-inflammatory activity by inhibiting the expression of interleukin 6 (IL-6), cyclooxygenase 2 (COX-2), tumor necrosis factor α (TNF-α), and nuclear factor-kappa B (NF-kB) in tetradecanoylphorbol acetate (TPA)-induced inflammation models (Luo et al., 2019; Zhang et al., 2020b). Moreover, these essential oils also demonstrate high antioxidant, antibacterial, and antifungal activities (Luo et al., 2019). The essential oil of *A. rugosa* (AREO), mainly composed of methyleugenol, estragole, and eugenol, exhibits strong pesticide activity against *Meloidogyne incognita*, with a LC_50_ value of 47.3 μg/mL (Li et al., 2013). ROEO also shows suppression of the lipopolysaccharide (LPS)-induced inflammation by inhibiting the expression of COX-2 and inducible nitric oxide synthase (iNOS) and blocking the production of TNF-α (Yu et al., 2013). Lavender essential oils have been used cosmetically and therapeutically for centuries, and their biological activities have been extensively studied (Cavanagh and Wilkinson, 2002). Borges et al. (2019) indicated that ROEO possesses strong anti-inflammatory activity and can be used as a remedy for inflammatory diseases (Borges et al., 2019). Though the essential oil extracted from *Salvia japonica* Thunb. (SJEO) has been widely used, little is known about its biological activity. Herb essential oils exert their diverse biological activities by acting on various pathways using different chemical components. The composition and bioactivity of essential oils extracted from Lamiaceae plants have been analyzed in many studies; however, their anti-inflammatory, antioxidant, antitumor, and anti-arthritis activities need to be systematically explored from multiple aspects (Nikolić et al., 2014; Vyry Wouatsa et al., 2014; Park et al., 2016; Mamadalieva et al., 2017, 2019; Mouahid et al., 2017; Borges et al., 2019; Karpinski, 2020).

The chemical composition of plant essential oil is influenced by numerous factors, such as the growing environment, harvest time, and plant organ used for essential oil extraction. Therefore, it is necessary to determine the phytochemical composition of the essential oil before carrying out further studies on their bioactivities. The current study aimed to elucidate the biological activity through the determination of the chemical composition of the essential oils extracted from six Lamiaceae plants with gas chromatography-mass spectrometry (GC-MS). The diversity of chemical components was analyzed by hierarchical cluster analysis in six essential oils from six Lamiaceae folk medicinal plants. Diverse biological activities, including anti-inflammatory, antitumor, and antioxidant activities, were evaluated through corresponding models. The model of complete Freund's adjuvant (CFA)-induced rheumatoid arthritis was used to evaluate the anti-inflammatory activity and related mechanisms of the six essential oils, and the LNCaP and B16 cell lines were used to estimate the antitumor activity. The 1,1-diphenyl-2-picrylhydrazyl (DPPH) free radical method was carried out to determine free radical scavenging capacity of these essential oils. Our study provides additional data to support the use of essential oils from Lamiaceae plants as a drug treatment.

## Materials and methods

### Plant materials and chemicals

The local name, storage locations, and collection dates of *A. rugosa, L. angustifolia, P. frutescens, P. cablin, R. officinalis*, and *S. japonica* samples are shown in Table 1. The leaves of *A. rugosa, P. cablin, P. frutescens, R. officinalis* were used for essential oil extraction, while the aerial parts of *L. angustifolia* and *S. japonica* were used for essential oils extractions. All plant samples were confirmed by Professor Nian Liu of Zhongkai University of Agriculture and Engineering (Guangzhou, China). All chemicals used in this study were purchased from Aladdin Reagent Inc. (Shanghai, China).

**Table 1:** Latin name, local name, voucher specimen number, and collection time of six Lamiaceae plants.

**Latin name**	**Local name**	**Voucher number**	**Collection time**	**Storage location**
*Pogostemon cablin* (Blanco) Benth.	Guanghuoxiang	2018-100C	2018.10	Institute of Natural Medicine & Green Chemical, School of Chemical Engineering and Light Industry, Guangdong University of Technology
*Salvia japonica* Thunb.	Shuweicao	2018-102C	2018.08	
*Perilla frutescens* (L.) Britton	Zisuye	2018-106C	2018.07	
*Rosmarinus officinalis* L.	Midiexiang	2018-103C	2018.09	
*Lavandula angustifolia* Mill.	Xunyicao	2018-101C	2018.12	
*Agastache rugosa* (Fisch. & C. A. Mey.) Kuntze	Huoxiang	2018-105C	2018.11	

### Essential oil extraction

The steam distillation method was used to extract essential oils from the six plant samples as previously described (Zhang et al., 2020b). The plants were cleaned, ground, passed through a 0.45-mm sieve, and distilled with a steam distillation device for 3.5 h (Zhang et al., 2017b). The isolated essential oils were dried as previously described in a former research and stored in individual brown glass bottles at 4 °C until use (Xiang et al., 2017).

### GC-MS analysis

The phytochemical compositions of PCEO, PFEO, SJEO, ROEO, LAEO, and AREO were identified according to previous methods (Xiang et al., 2017; Zhang et al., 2017b) using a GC-MS system with a DB-5MS capillary column (0.25 mm × 30 m, i.d. 0.25 μm) (Agilent, Santa Clara, CA, USA). The carrier gas was Helium (He) at a flow rate of 1 mL/min. The initial temperature was set as 40°C for 1 min, then increased to 280°C by 3°C/min, and held at 280°C for 5 min. The split ratio was set as 100:1. For MS conditions, the ionization conditions were as follows: pressure of 50 kPa, electron energy of 70 eV, and ion source temperature of 200°C.

Each component of essential oils was determined based on the retention index (RI), which was calculated using a series of n-alkanes (C_6_-C_40_) (Xue et al., 2016). Additionally, the mass spectrum of each compound was searched against the NIST Standard Reference Database (NIST Chemistry WebBook, 2014, over 40,000 compounds in this database) and databases published elsewhere (Zhang et al., 2017a). The total ionization chromatography (TIC) was obtained and used for determining the contents of each compound (Supplementary Figure S1).

### Animals

The animal experiments were carried out following ethical guidelines of the Laboratory Animal Center of Sun Yat-sen University. Male rats (6–8 weeks old, 210 ± 30 g body weight) were purchased from Sun Yat-sen University and raised under normal conditions (25 ± 2°C, 12/12 h light/dark cycle). Food and water were fed as required during the experiment.

### Experimental treatments of adjuvant arthritis

According to our previous study, the rats were acclimatized for 7 days and then divided into 10 groups, with 10 rats in each group: (a) normal control (Con) group, (b) model (CFA) group, (c) negative control (NC) group, (d) positive control (PC) group, (e) PCEO group, (f) PFEO group, (g) ROEO group, (h) SJEO group, (i) LAEO group, and (j) AREO group (Zhang et al., 2020a).

Rats in the Con. group were not given any treatments. In the model group, CFA (0.1 mL) was subcutaneously injected into each rat after routine sterilization from Day 8 to Day 21 to induce arthritis. Then these rats were raised under normal conditions until use. Tween 80 was given to the rats in the NC group, while ibuprofen (100 mg/kg, dissolved in Tween 80) was given to those in the PC group from Day 8 to Day 20 (Khayyal et al., 2005). Rats in PCEO, PFEO, ROEO, SJEO, LAEO, and AREO groups were treated with corresponding essential oils (100 mg/kg, dissolved in Tween 80) from Day 8 till the end of the experiment. The arthritis score was recorded from Day 8 to Day 20 for all groups. The 5-point method was used to assess and grade the severity of the swelling, erythema, or stiffness in the paw: 0 = no signs of illness; 1 = mild swelling and erythema in the ankle/wrist; 2 = swelling and erythema in the ankle/wrist; 3 = severe swelling and erythema in the ankle/wrist; and 4 = severe illness in the paw or front leg. Both the hind feet were graded, and the total score was not allowed to exceed 8 (Funk et al., 2010). The rats were then sacrificed and their ankle joints were sampled and stored in 4% (v/v) paraformaldehyde for subsequent histological analysis.

### Histological analysis and immunohistochemical staining

As described previously (Zhang et al., 2020a), the ankle joints were paraffin-embedded and the sections were observed using light microscopy (Olympus IX71, OLYMPUS, Japan). The IL-1, IL-6, COX-2, and TNF-α antibodies, all diluted at a 1:200 ratio, were used for immunohistochemical staining. The number of positive cells was counted with ImageJ using photos taken with a fluorescence microscope (NIH, Bethesda, MD, USA).

For immunohistochemical analysis, the sections were incubated overnight with IL-1β (dilution 1:200), IL-6 (dilution 1:200), COX-2 (dilution 1:200), and TNF-α (dilution 1:200) antibodies at 4°C and then treated with a secondary antibody and alkaline phosphatase-labeled streptavidin (1:200) at 25°C for up to 1 h. Sections were developed with 3,30-diaminobenzidine (DAB) solution. Image analysis software (Image-Pro Plus) was used to count the number of positive cells, and these were observed using a fluorescence microscope (NIH, Bethesda, MD) (Zhang et al., 2020b).

### Determination of antitumor activity

Anittumor activity of essential oil was investigated via human prostate cancer cell model LNCap and mouse B16 melanoma cell lines *in vitro*. In PCEO, PFEO, ROEO, SJEO, LAEO, and AREO groups, the cytotoxicity of essential oils on LNCaP and B16 cells treated with essential oils was assessed using MTT [3 - (4,5 - dimethyl - 2 - thiazolyl) - 2,5 - diphenyltetrazolium bromide] values described in Zhang et al. (2020a). The B16 and LNCaP cells were cultured in DMEM and RPMI1640, respectively, supplemented with 10% FBS, 2 mM glutamine, 100 mg/mL streptomycin, and 100 U/mL penicillin. The cells were maintained in a humidified 5% CO_2_ incubator at 37 °C and were subcultured every 3–4 days to maintain logarithmic growth and allowed to grow for 24 h before the treatments were applied. The cells were then treated with different concentrations of the essential oil (**Table 3**), and then the absorbance at 570 nm was read on a microplate reader. The IC_50_ value of MTT assays is defined as essential oils concentration resulting in a 50% reduction of absorbance (Kanipandian and Thirumurugan, 2014). Hydroquinone and Paclitaxel were used as positive controls for B16 and LNCaP cells, respectively (Zhang et al., 2017c; Rodboon et al., 2020).

### Determination of antioxidant activity

The antioxidant activity of the six essential oils was evaluated by DPPH free radical scavenging capacity (Xiang et al., 2017; Zhang et al., 2020a). DPPH solution (67 μg/mL) was mixed with each of the essential oils at various concentrations and incubated at 25°C for 30 min in the dark. The absorbance was then read at 517 nm. The scavenging percentage was calculated as follows:


$$\begin{array}{ll} \text{Scavenging percentage}\left(\text{\%}\right)\\ = & \left[1-\left(A_{sample}--A_{\text{blank}}\right)/A_{control}\right]\times \;100 \end{array}$$

### Statistical analysis

One-way analysis of variance (ANOVA) was used to test statistical significance, and the result was considered significant at *P* ≤ 0.05. Data were presented as mean ± standard deviation (SD). Based on the content of each component, hierarchical cluster analysis (HCA) was performed for chemical compositions in the six essential oils using pheatmap package (version 1.0.12).

## Results and discussion

### Phytochemical compositions of the six essential oils

**Figure 1:**
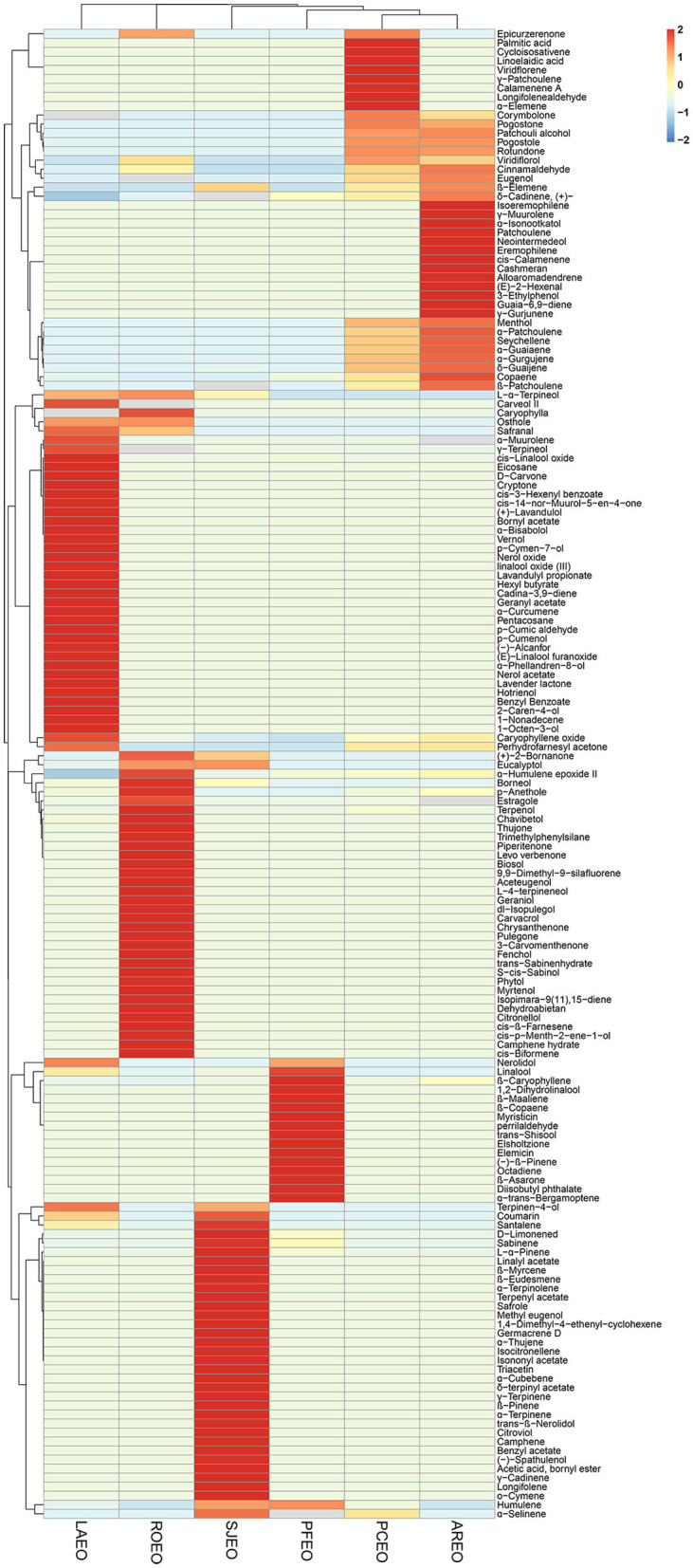
Hierarchical cluster analysis for the chemical compositions in six essential oils.

A total of 167 components were identified from six essential oils (Table 2) using GC-MS. Based on the species and quantity of compositions in each essential oil, the relationship of the six Lamiaceae folk medicinal plants was analyzed via hierarchical cluster. The results showed that the six essential oils were clearly divided into six classes, and the six essential oils have their unique principal components (Figure 1). The proportions of corymbolone, pogostone, patchouli alcohol, pogostole, rotundone, menthol, α-patchoulene, seychellene, α-guaiaene, α-gurgujene, and δ-guaijene showed similarity in PCEO and AREO. The proportions of L-α-terpineol, osthole, and safranal showed similarity in LAEO and ROEO. The proportions of nerolidol and linalool showed similarity in LAEO and PFEO. The proportion of terpinen-4-ol showed similarity in LAEO and SJEO. The proportions of (+)-2-bornanone and eucalyptol showed similarity in SJEO and ROEO. The proportion of humulene showed similarity in SJEO and PFEO. The proportion of α-selinene showed similarity in SJEO and PCEO. The proportion of epicurzerenone showed similarity trend in PCEO and ROEO (Figure 1). β-Caryophyllene was present in all six essential oils, while patchouli alcohol and caryophyllene oxide were found in all essential oils except PFEO. In summary, PCEO has the latest relationship with AREO, followed by PFEO, SJEO, ROEO, and LAEO. This relationship was also partly proved by the biological activities of six essential oils in the following results.

**Table 2:** Chemical compositions of six Lamiaceae plants.

**No**	**Compounds ^i^**	**RI2 ^ii^**	**RI3 ^iii^**	**Ref**	**Cas**	**Relative content (%)** ^**iv**^					
						**PCEO**	**PFEO**	**SJEO**	**ROEO**	**LAEO**	**AREO**
1	Menthol	1181		b	015356-70-4	0.22	-	-	-	-	-
2	Terpenol	1200	1010	c	000098-55-5	0.28	-	-	3.36	-	-
3	Cinnamaldehyde	1283	1277		014371-10-9	1.41	-	-	0.99	0.18	2.05
4	p-Anethole	1291	1289	d	000104-46-1	0.49	-	-	3.73	0.48	0.92
5	p-Allylguaiacol/Eugenol	1370	1373	b	000097-53-0	5.64	-	-	-	-	8.29
6	Copaene	1394	1394	b	003856-25-5	0.68	0.18	-	-	0.09	1.34
7	β-Patchoulene	1405	1406	b	000514-51-2	0.19	-	-	-	-	0.40
8	β-Elemene	1407	1382	a	000515-13-9	0.20	-	0.24	-	-	0.33
9	β-Caryophyllene	1443	1441	b	000087-44-5	1.17	7.76	1.04	0.68	0.81	2.12
10	α-Guaiaene	1457	1455		003691-12-1	1.04	-	-	-	-	1.62
11	Seychellene	1472	1375	c	020085-93-2	0.86	-	-	-	-	1.34
12	Humulene	1478	1364	a	006753-98-6	0.22	0.92	0.87	-	0.13	-
13	α-Patchoulene	1485	1288	c	000560-32-7	0.47	-	-	-	-	0.76
14	α-Elemene	1488	1348	c	005951-67-7	0.12	-	-	-	-	-
15	γ-Patchoulene	1492	1424	a	000508-55-4	0.14	-	-	-	-	-
16	α-Selinene	1511	1587	c	000473-13-2	0.14	-	0.25	-	-	-
17	α-Gurgujene	1519	1519		000489-40-7	0.75	-	-	-	-	1.09
18	δ-Guaijene	1527	1525		003691-11-0	2.39	-	0.16	-	-	3.48
19	δ-Cadinene, (+)-	1541	1547		000483-76-1	0.35	0.28	-	0.13	-	0.60
20	Calamenene A	1544	1543		000483-77-2	0.15	-	-	-	-	-
21	Caryophyllene oxide	1613	1613	b	001139-30-6	1.22	-	0.21	0.72	4.31	1.94
22	Epicurzerenone	1629	1623		020085-85-2	0.16	-	-	0.14	-	-
23	α-Humulene epoxide II	1640	1615		019888-34-7	0.18	0.16	0.12	0.53	-	0.23
24	Viridiflorol	1654	1620		000552-02-3	1.29	-	-	0.91	-	1.02
25	Viridflorene	1672	1656		021747-46-6	0.40	-	-	-	-	-
26	Pogostole	1688	1655	b	021698-41-9	3.96	-	-	-	-	3.94
27	Patchouli alcohol	1707	1587	a	005986-55-0	43.04	-	0.12	0.26	0.20	45.70
28	Rotundone	1736	1722		018374-76-0	1.59	-	-	-	-	1.53
29	Pogostone	1743	1641	a	023800-56-8	14.35	-	-	-	-	11.92
30	Longifolenealdehyde	1755	1668		019890-84-7	0.12	-	-	-	-	-
31	Cycloisosativene	1766	1530		022469-52-9	0.31	-	-	-	-	-
32	Perhydrofarnesyl acetone	1850	1848		000502-69-2	0.12	-	-	-	0.23	0.13
33	Corymbolone	1922	1899		097094-19-4	0.31	-	-	-	-	0.20
34	Palmitic acid	1962	1961		000057-10-3	0.16	-	-	-	-	-
35	Linoelaidic acid	2142			000506-21-8	0.16	-	-	-	-	-
36	L-α-Pinene	939	945	e	007785-26-4	-	0.50	5.97	-	-	-
37	Sabinene	979	977		003387-41-5	-	0.23	0.96	-	-	-
38	(-)-beta-Pinene	985	1010	e	018172-67-3	-	0.15	-	-	-	-
39	D-Limonened	1035	1100	e	005989-27-5	-	0.69	4.13	-	-	-
40	cis-Linalool oxide	1079	1078		005989-33-3	-	0.24	-	-	5.69	-
41	Linalool	1111	1230	e	000078-70-6	-	67.65	8.89	1.56	29.84	-
42	1,2-Dihydrolinalool	1137	1125	b	018479-51-1	-	0.93	-	-	-	-
43	3,7-Octadiene-2,6-diol, 2,6-dimethyl-, (3E)-	1195	1191		051276-34-7	-	0.15	-	-	-	-
44	Elsholtzione	1208	1454		000488-05-1	-	0.22	-	-	-	-
45	trans-Shisool	1282	1248		022451-48-5	-	0.24	-	-	-	-
46	perrilaldehyde	1290	1632	e	002111-75-3	-	1.45	-	-	-	-
47	α-trans-Bergamoptene	1499	1490		013474-59-4	-	1.60	-	-	-	-
48	β-Copaene	1504	1460	b	018252-44-3	-	0.32	-	-	-	-
49	β-Maaliene	1518	1418		000489-29-2	-	0.16	-	-	-	-
50	Myristicin	1536	1382	g	000607-91-0	-	0.29	-	-	-	-
51	Elemicin	1562	1560	b	000487-11-6	-	0.15	-	-	-	-
52	Nerolidol	1572	1564	f	000142-50-7	-	0.27	-	-	-	-
53	β-Asarone	1687	1641	b	005273-86-9	-	0.57	-	-	-	-
54	Diisobutyl phthalate	1881	1873		000084-69-5	-	0.90	-	-	-	-
55	Isocitronellene	919	917		085006-04-8	-	-	0.21	-	-	-
56	α-Thujene	930	931	b	002867-05-2	-	-	0.21	-	-	-
57	Camphene	956	957	b	000079-92-5	-	-	0.83	-	-	-
58	β-Pinene	984	1023	k	000127-91-3	-	-	1.01	-	-	-
59	β-Myrcene	992	988	b	000123-35-3	-	-	0.61	-	-	-
60	o-Cymene	1031	1029	b	000527-84-4	-	-	1.31	-	-	-
61	Eucalyptol	1040	1039	b	000470-82-6	-	-	6.15	6.05	0.40	0.11
62	α-Terpinolene	1095	10.94	b	000586-62-9	-	-	0.16	-	-	-
63	(+)-2-Bornanone	1160	1144	k	000464-49-3	-	-	9.54	15.10	-	-
64	Benzyl acetate	1172	1168	b	000140-11-4	-	-	5.73	-	-	-
65	Isononyl acetate	1177	1180		058430-94-7	-	-	0.42	-	-	-
66	Borneol	1180	1178	b	000507-70-0	-	-	3.20	14.58	1.84	-
67	Terpinen-4-ol	1189	1188		000562-74-3	-	-	0.84	-	1.03	-
68	L-α-Terpineol	1201	1514	k	010482-56-1	-	-	2.33	4.90	4.28	-
69	γ-Terpineol	1207	1217	b	000586-81-2	-	-	0.32	-	11.11	-
70	Linalyl acetate	1259	1262	b	000115-95-7	-	-	3.26	-	0.12	-
71	1,4-Dimethyl-4-ethenyl-cyclohexene	1290	949		001743-61-9	-	-	1.22	-	-	-
72	Acetic acid, bornyl ester	1298			092618-89-8	-	-	1.47	-	-	-
73	Safrole	1302	1288	b	000094-59-7	-	-	2.65	-	-	-
74	γ-Terpinene	1309	1269	b	000099-85-4	-	-	0.23	-	-	-
75	δ-terpinyl acetate	1327	1316		093836-50-1	-	-	0.22	-	-	-
76	Triacetin	1353	1344	h	000102-76-1	-	-	6.84	-	-	-
77	Terpenyl acetate	1362	1367	j	000080-26-2	-	-	10.52	-	-	-
78	α-Terpinene	1364	1243	k	000099-86-5	-	-	2.57	-	-	-
79	α-Cubebene	1394	1382	b	017699-14-8	-	-	0.28	-	-	-
80	Methyl eugenol	1410	1410		000093-15-2	-	-	0.13	-	-	-
81	Longifolene	1433	1427	b	000475-20-7	-	-	0.67	-	-	-
82	Santalene	1436	1431		000512-61-8	-	-	0.50	-	0.17	-
83	Citroviol	1439	1413		000128-51-8	-	-	3.79	-	-	-
84	Coumarin	1461	1458	b	000091-64-5	-	-	0.55	-	0.34	-
85	Germacrene D	1505	1508	b	023986-74-5	-	-	0.26	-	-	-
86	β-Eudesmene	1512	1507		017066-67-0	-	-	0.26	-	-	-
87	γ-Cadinene	1536	1534	b	039029-41-9	-	-	0.06	-	-	-
88	trans-β-Nerolidol	1573	1567		040716-66-3	-	-	0.88	-	-	-
89	(-)-Spathulenol	1605	1619		077171-55-2	-	-	0.25	-	-	-
90	α-Curcumene	1555	1499	h	000644-30-4	-	-	-	-	0.10	-
91	trans-Sabinenhydrate	1106	1106		017699-16-0	-	-	-	0.11	-	-
92	Fenchol	1124	1125		001632-73-1	-	-	-	0.24	-	-
93	cis-p-Menth-2-ene-1-ol	1131	1129		029803-82-5	-	-	-	0.46	-	-
94	Chrysanthenone	1135	1126		000473-06-3	-	-	-	0.14	-	-
95	Camphene hydrate	1163	1150		000465-31-6	-	-	-	0.25	-	-
96	dl-Isopulegol	1168	1167	b	050373-36-9	-	-	-	0.20	-	-
97	L-4-terpineneol	1189	1182		020126-76-5	-	-	-	2.77	-	-
98	Trimethylphenylsilane	1196			000768-32-1	-	-	-	0.26	-	-
99	Estragole	1207	1201		000140-67-0	-	-	-	0.18	-	-
100	Myrtenol	1211	1201	b	000515-00-4	-	-	-	0.54	-	-
101	S-cis-Sabinol	1217	1179	b	003310-02-9	-	-	-	0.25	-	-
102	Levo verbenone	1228	1204	i	001196-01-6	-	-	-	15.29	-	-
103	Citronellol	1231	1230		000106-22-9	-	-	-	0.78	-	-
104	Pulegone	1252	1250	b	000089-82-7	-	-	-	0.19	-	-
105	Thujone	1253	1117		000546-80-5	-	-	-	0.65	-	-
106	Geraniol	1258	1255		000106-24-1	-	-	-	0.90	-	-
107	3-Carvomenthenone	1268	1268		000089-81-6	-	-	-	0.43	-	-
108	Biosol	1288	1332		003228-02-2	-	-	-	0.68	-	-
109	Carvacrol	1306	1306		000499-75-2	-	-	-	0.63	-	-
110	Piperitenone	1358	1348		000491-09-8	-	-	-	0.51	-	-
111	Chavibetol	1372	1362	l	000501-19-9	-	-	-	17.72	1.32	-
112	Safranal	1401	1596		000116-26-7	-	-	-	0.30	0.44	-
113	cis-β-Farnesene	1462	1434		028973-97-9	-	-	-	0.11	-	-
114	Aceteugenol	1534	1525		000093-28-7	-	-	-	0.17	-	-
115	Caryophylla-4(12),8(13)-dien-5-beta-ol	1664	1644		019431-80-2	-	-	-	0.26	-	-
116	9,9-Dimethyl-9-silafluorene	1700			013688-68-1	-	-	-	0.17	-	-
117	Isopimara-9(11),15-diene	1954	1920		039702-28-8	-	-	-	0.11	-	-
118	cis-Biformene	2045	2004		005957-33-5	-	-	-	0.15	-	-
119	Dehydroabietan	2095	2057		019407-28-4	-	-	-	0.11	-	-
120	Phytol	2119	2122		000150-86-7	-	-	-	0.11	-	-
121	Osthole	2166	2174		000484-12-8	-	-	-	0.45	0.43	-
122	1-Octen-3-ol	978	972		003391-86-4	-	-	-	-	0.25	-
123	Lavender lactone	1045	1046		001073-11-6	-	-	-	-	0.15	-
124	(E)-Linalool furanoxide	1095	1094		034995-77-2	-	-	-	-	4.10	-
125	Hotrienol	1110	1108		029957-43-5	-	-	-	-	0.88	-
126	(-)-Alcanfor	1158	1146		000464-48-2	-	-	-	-	0.56	-
127	Nerol oxide	1160	1164		001786-08-9	-	-	-	-	0.43	-
128	(+/-)-Lavandulol	1172	1170		058461-27-1	-	-	-	-	3.38	-
129	α-Phellandren-8-ol	1177	1181		001686-20-0	-	-	-	-	0.35	-
130	linalool oxide (III)	1181	1199		039028-58-5	-	-	-	-	0.38	-
131	Hexyl butyrate	1192	1195		002639-63-6	-	-	-	-	0.19	-
132	Cryptone	1199	1184		000500-02-7	-	-	-	-	1.11	-
133	Carveol II	1228	1231		001197-06-4	-	-	-	-	0.21	-
134	p-Cumenol	1231	1247		000099-89-8	-	-	-	-	0.10	-
135	Vernol	1234	1233		000106-25-2	-	-	-	-	0.85	-
136	p-Cumic aldehyde	1253	1249		000122-03-2	-	-	-	-	0.47	-
137	D-Carvone	1255	1225		002244-16-8	-	-	-	-	0.26	-
138	Lavandulyl propionate	1293	1375		059550-34-4	-	-	-	-	6.42	-
139	Bornyl acetate	1297	1286		000076-49-3	-	-	-	-	0.29	-
140	p-Cymen-7-ol	1300	1302		000536-60-7	-	-	-	-	0.43	-
141	Nerol acetate	1366	1347		000141-12-8	-	-	-	-	1.09	-
142	Geranyl acetate	1385	1386		000105-87-3	-	-	-	-	1.78	-
143	2-Caren-4-ol	1492	1181		006617-35-2	-	-	-	-	0.22	-
144	α-Muurolene	1518			031983-22-9	-	-	-	-	0.11	-
145	Cadina-3,9-diene	1541	1529		000523-47-7	-	-	-	-	0.12	-
146	cis-3-Hexenyl benzoate	1584	1550		025152-85-6	-	-	-	-	0.26	-
147	α-Bisabolol	1702	1701		000515-69-5	-	-	-	-	0.12	-
148	cis-14-nor-Muurol-5-en-4-one	1721	1696		063180-33-6	-	-	-	-	0.16	-
149	Benzyl Benzoate	1787	1789		000120-51-4	-	-	-	-	0.11	-
150	Nerolidol	2040	2030		007212-44-4	-	-	-	-	0.31	-
151	1-Nonadecene	2280	1960		018435-45-5	-	-	-	-	0.18	-
152	Eicosane	2490			000112-95-8	-	-	-	-	0.29	-
153	Pentacosane	2499			000629-99-2	-	-	-	-	0.10	-
154	Menthol	1181	1171		001490-04-6	-	-	-	-	-	0.29
155	Guaia-6,9-diene	1489	1450		036577-33-0	-	-	-	-	-	0.21
156	Patchoulene	1492	1485		001405-16-9	-	-	-	-	-	0.22
157	γ-Muurolene	1495	1483		030021-74-0	-	-	-	-	-	0.18
158	Alloaromadendrene	1511	1495		025246-27-9	-	-	-	-	-	0.27
159	cis-Calamenene	1544	1531		072937-55-4	-	-	-	-	-	0.27
160	Cashmeran	1592	1503		033704-61-9	-	-	-	-	-	1.25
161	3-Ethylphenol	1598			000620-17-7	-	-	-	-	-	1.84
162	α-Isonootkatol	1610			1380573-94-3	-	-	-	-	-	0.92
163	Neointermedeol	1664	1656		005945-72-2	-	-	-	-	-	0.39
164	Eremophilene	1669			010219-75-7	-	-	-	-	-	0.23
165	γ-Gurjunene	1673	1664		022567-17-5	-	-	-	-	-	0.16
166	(E)-2-Hexenal	1750			006728-26-3	-	-	-	-	-	1.21
167	Isoeremophilene	1765	1721		004630-07-3	-	-	-	-	-	0.43
	Total/%					84.29	86.01	92.47	98.76	89.21	98.92

*Retention index (RI) and relative content of identified compounds in six essential oils*.

*^a^ Chen et al. (2017); ^b^ Luo et al. (2019); ^c^ Zhang et al. (2019); ^d^ Vieira et al. (2019); ^e^ Li (2010); ^f^ Xue et al. (2016); ^g^ Gao and Zhang (2019); ^h^ Zhang et al. (2017a); ^j^ Mouahid et al. (2017); ^k^ Li et al. (2016); ^l^ Vyry Wouatsa et al. (2014).*

*^i^ Compound listed in the order of elution from methyl silicone capillary column.*

*^ii^ Retention index relative to n-alkanes (C_6_-C_40_) on the same methyl silicone capillary column.*

*^iii^ Literature indices.*

*^iv^ PFEO, the essential oils of Perilla frutescens (L.) Britton; PCEO, the essential oils of Pogostemon cablin (Blanco) Benth.; SJEO, the essential oils of Salvia japonica Thunb.; ROEO, the essential oils of Rosmarinus officinalis L.; LAEO, the essential oils of Lavandula angustifolia Mill.; AREO, the essential oils of Agastache rugosa (Fisch. & C. A. Mey.) Kuntze*.

**Figure 2:**
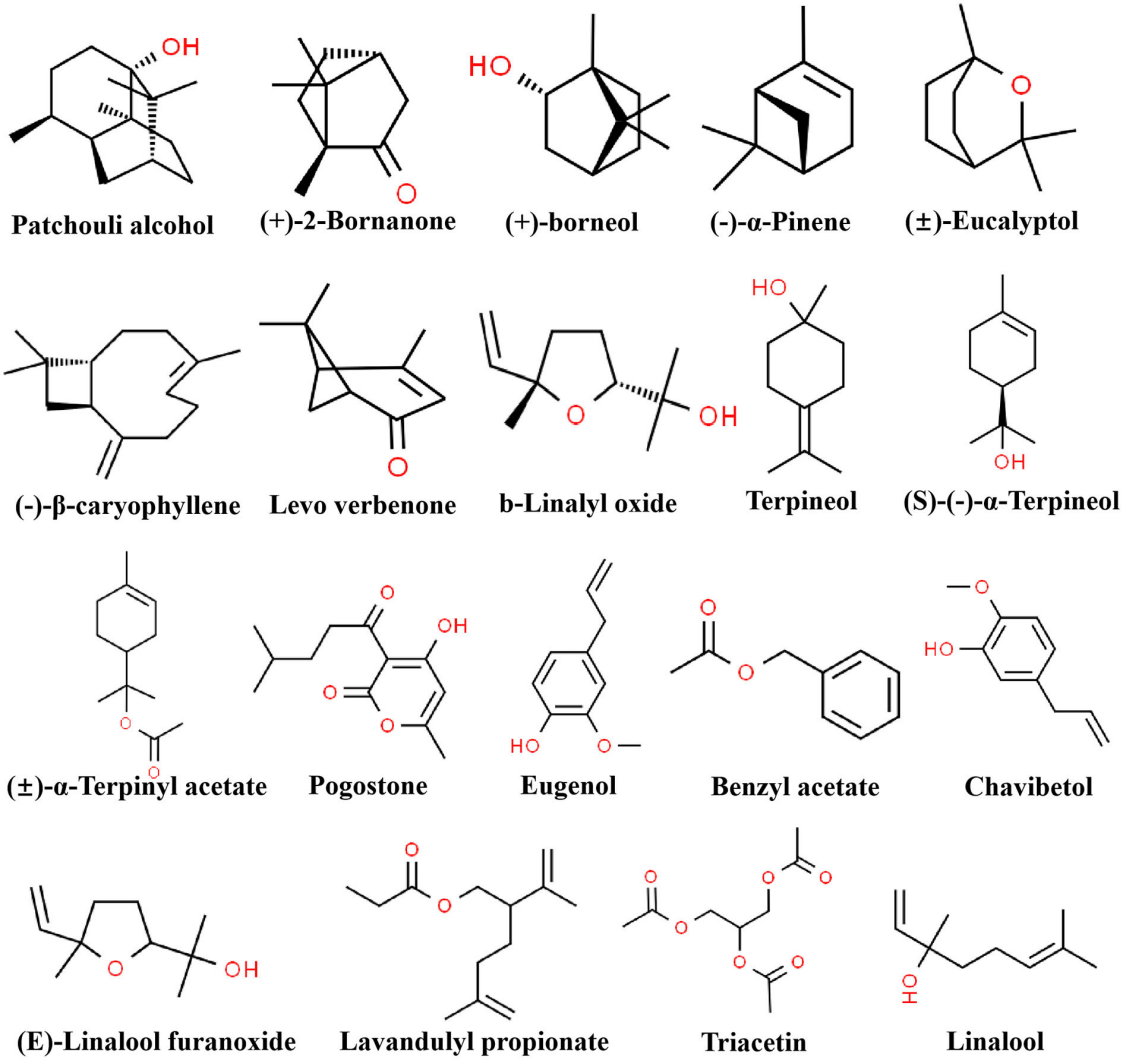
The chemical structure of 15 components in six essential oils. The relative content of each component was more than 4%.

To further investigate the difference of the six essential oils, the major compounds of essential oils were compared in the analysis. The total relative contents of PCEO, PFEO, ROEO, SJEO, LAEO, and AREO were 84.29, 86.01, 98.76, 92.47, 89.21, and 98.92%, respectively. The dominant components of PCEO were patchouli alcohol (43.04%), pogostone (14.35%), and p-allylguaiacol/eugenol (5.638%). The major components of PFEO were linalool (67.65%) and β-caryophyllene (7.7564%). The main components of SJEO were terpenyl acetate (10.52%), (+)-2-bornanone (9.54%), camphor (9.54%), linalool (8.89%), L-α-pinene (5.97%), triacetin (6.84%), eucalyptol (6.15%), plastolin I (5.73%), and _D_-limonened (4.13%). The principal components of ROEO were chavibetol (17.72%), verbenone (15.29%), camphor (15.10%), borneol (14.58%), eucalyptol (6.05%), and α-terpineol (4.90%). The key components of LAEO were linalool (29.84%), γ-terpineol (11.11%), caryophyllene oxide (4.31%), *cis*-linalool oxide (5.69%), L-α-terpineol (4.28%), (E)-linalool furanoxide (4.10%), and lavandulyl propionate (6.42%). The primary components of AREO were patchouli alcohol (45.70%), pogostone (11.92%), and eugenol (8.29%). Linalool was found in LAEO, PFEO, ROEO, and SJEO, exhibiting the highest relative content in PFEO (67.66%), followed by LAEO (29.84%), SJEO (8.89%), and ROEO (1.56%). ROEO and AREO shared many common components, including patchouli alcohol, pogostone, and eugenol. In POEO and AREO, the relative contents of patchouli alcohol, pogostone, and eugenol were 43.04, 14.35, and 5.64%; and 45.70, 11.92, and 8.29%, respectively (Table 2). Meanwhile, the chemical structures of 15 components are shown in Figure 2 to provide more reference for later researchers, including patchouli alcohol, eugenol,β-caryophyllene, pogostone, L-α-pinene, *cis*-linalool oxide, linalool, eucalyptol, (+)-2-bornanone, benzyl acetate, (+)-borneol, L-α-terpineol, γ-terpineol, triacetin, terpenyl acetate, levo verbenone, chavibetol, (E)-linalool furanoxide, and lavandulyl propionate. All 15 components were picked up according to their content, the and content was more than 4 %.

The chemical composition of essential oil is influenced by various factors. Some components have been previously reported in the essential oils of Lamiaceae species. For example, PFEO is proposed to mainly contain linalool (46.55%) and 2-hexanoylfuran (30.79%); ROEO is composed of α-pinene (45.35%) and D-limonene (18.42%); PCEO mainly contains patchouli alcohol (28.27%), α-bulnesene (18.29%), and α-guaiene (14.53%); LAEO mainly includes isononyl acetate (22.52%), α-pinene (11.47%), and benzyl acetone (10.93%); and SJEO mainly contains *o*-cymene (41.20%), (Z, E)-α-farnesene (10.82%), and γ-muurolene (9.89%) (Luo et al., 2019).

Our results indicated that the six Lamiaceae essential oils have diverse chemical compositions, and they could serve as good sources of eugenol, patchouli alcohol, linalool, eucalyptol, β-caryophyllene, terpenyl acetate, chavibetol, camphor, γ-terpineol, borneol, and α-pinene. Some of these essential oils have been reported to demonstrate promising antioxidant, anti-nociceptive, anti-cardiotoxicity, anti-cancer, and anti-inflammatory activities (Sharma et al., 1994; Santos and Rao, 2000; Khan et al., 2014; Vyry Wouatsa et al., 2014; Fidyt et al., 2016; Mamadalieva et al., 2017; Nieto, 2017; Lian et al., 2018; Liu et al., 2018; de Souza et al., 2019; Luo et al., 2019; Oner et al., 2019).

### Anti-arthritis activity of the six essential oils

**Figure 3:**
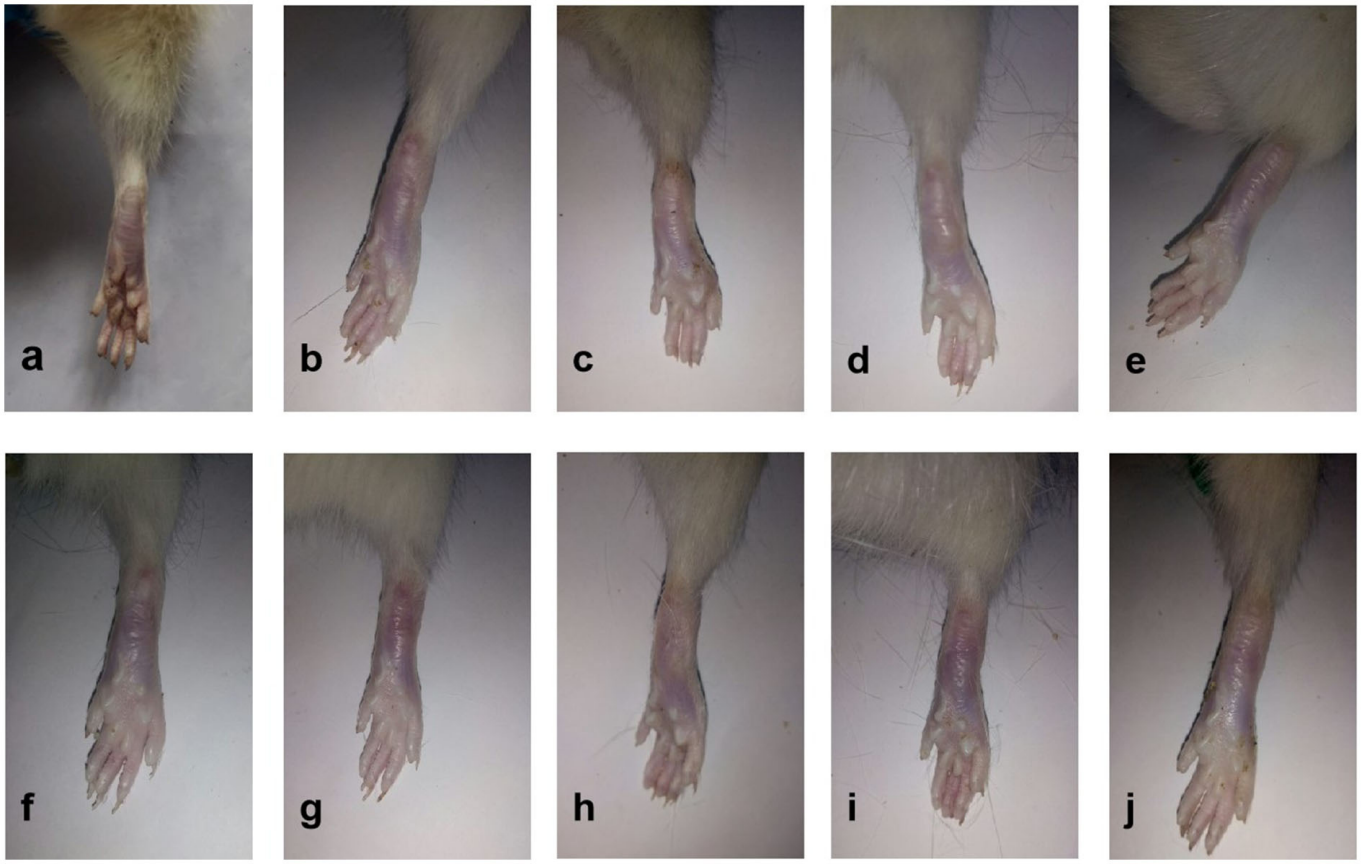
The appearance of rats with CFA-induced adjuvant arthritis. **(a)** Normal control (Con) group; **(b)** model (CFA) group; **(c)** negative control (NC) group; **(d)** positive control (PC) group; **(e)** PCEO group; **(f)** PFEO group; **(g)** ROEO group; **(h)** SJEO group; **(i)** LAEO group; and **(j)** AREO group.

As shown in Figure 3, all the six Lamiaceae essential oils displayed inhibitory effects on adjuvant arthritis in rats. Compared to the model group, PCEO, PFEO, ROEO, SJEO, LAEO, and AREO at a concentration of 100 mg/kg exhibited inhibitory effects on arthritis, among which PFEO manifested the highest anti-inflammatory activity, while PCEO showed the lowest. This result was consistent with that obtained from the PC group (ibuprofen treatment), which is effective for alleviating joint swelling in the rat models of arthritis.

**Figure 4:**
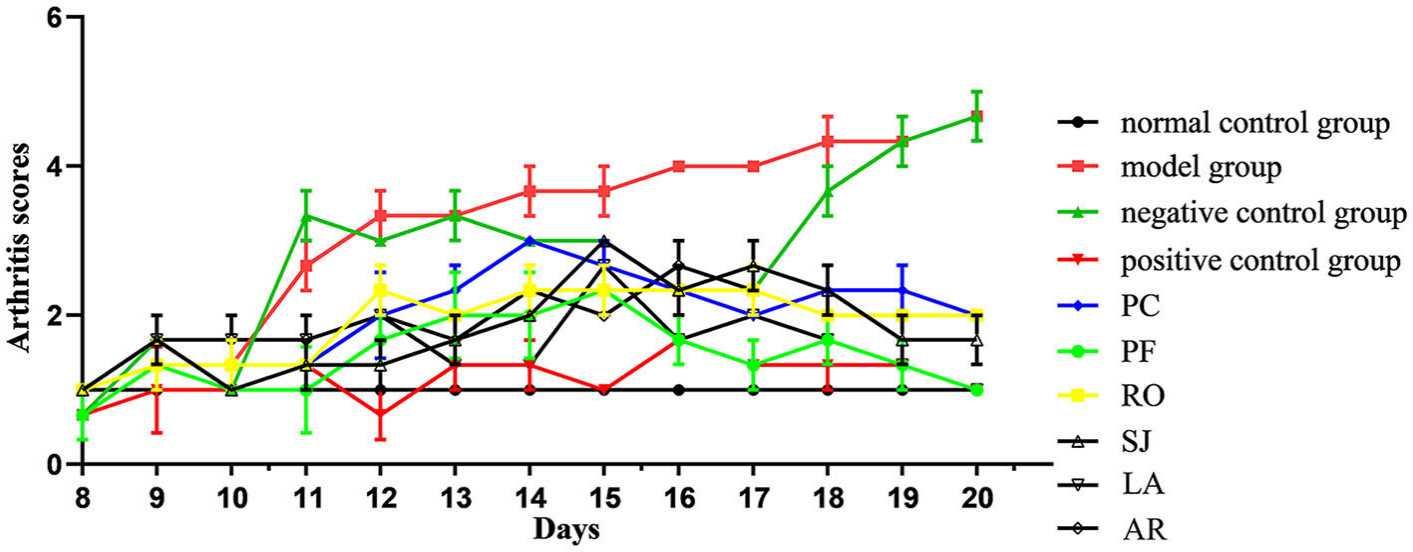
Arthritis scores of rats with CFA-induced adjuvant arthritis. The scores of normal control group, model (CFA) group, negative control (NC) group, positive control group, PCEO group (PC), PFEO group (PF), ROEO group (RO), SJEO group (SJ), LAEO group (LA), AREO group (AR) are shown with different color lines.

The ankle joints of rats in the model group were significantly swollen compared with those of the control (Figure 3). In addition, the arthritis score of the positive control group (ibuprofen treatment) was significantly lower than that of the model group (Figure 4), which implied the success of CFA-induced arthritis. The arthritis scores of six essential oil treatment groups were lower than that of the model group, with PL displaying the lowest score, which indicated that PFEO might possess the strongest anti-arthritis capacity (Figure 4).

**Figure 5:**
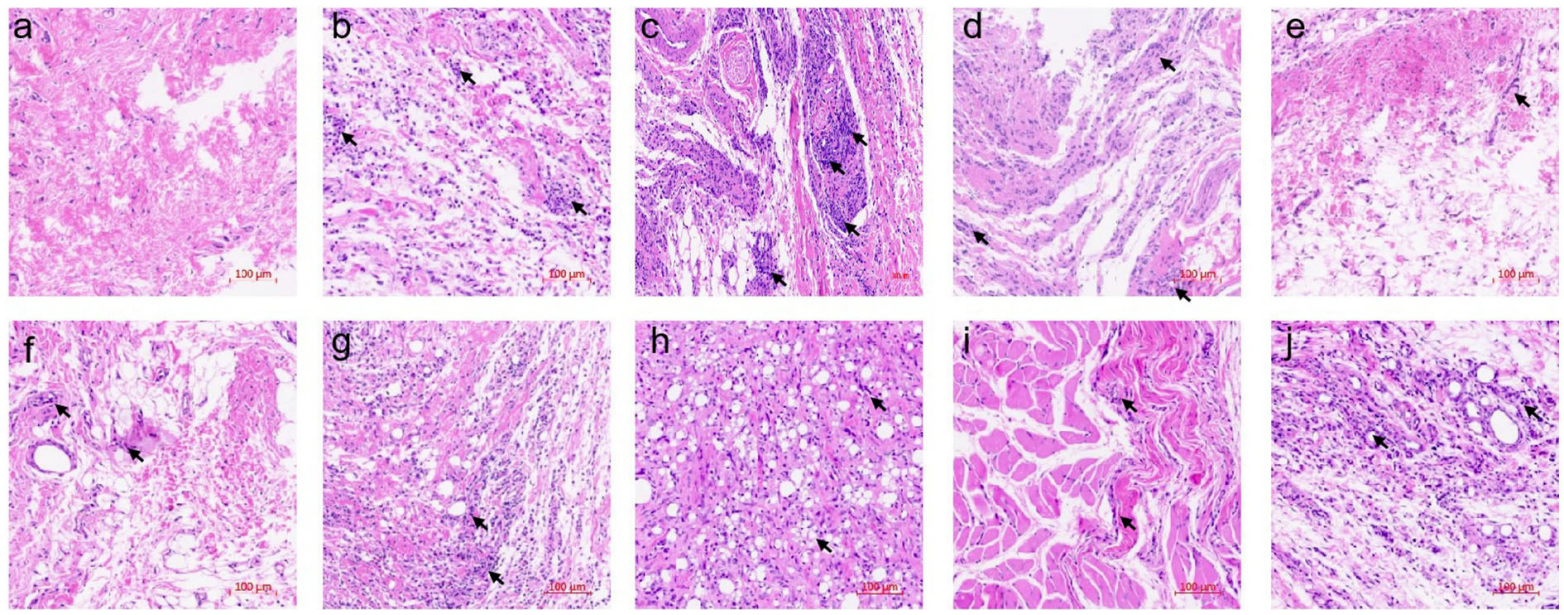
Histological sections of rat articular tissues showing severe cartilage damage, capillary hyperplasia, synovial proliferation, and lymphocyte infiltration (200 × magnification). **(a)** Normal control (Con) group; **(b)** model (CFA) group; **(c)** negative control (NC) group; **(d)** positive control (PC) group; **(e)** PCEO group; **(f)** PFEO group; **(g)** ROEO group; **(h)** SJEO group; **(i)** LAEO group; **(j)** AREO group. Arrows indicate the lesion sites.

To obtain further insight into the anti-arthritis effect of six essential oils, histological and immunohistochemical characterizations were conducted using articular tissues. Severe cartilage damage, capillary hyperplasia, synovial proliferation, and lymphocyte infiltration were observed in the model group, while lymphocyte infiltration and cartilage damage were significantly inhibited in the PC group (ibuprofen treatment) (Figure 5). PCEO, PFEO, ROEO, SJEO, LAEO, and AREO exhibited similar effects relative to ibuprofen, which may largely decrease the damage.

**Figure 6:**
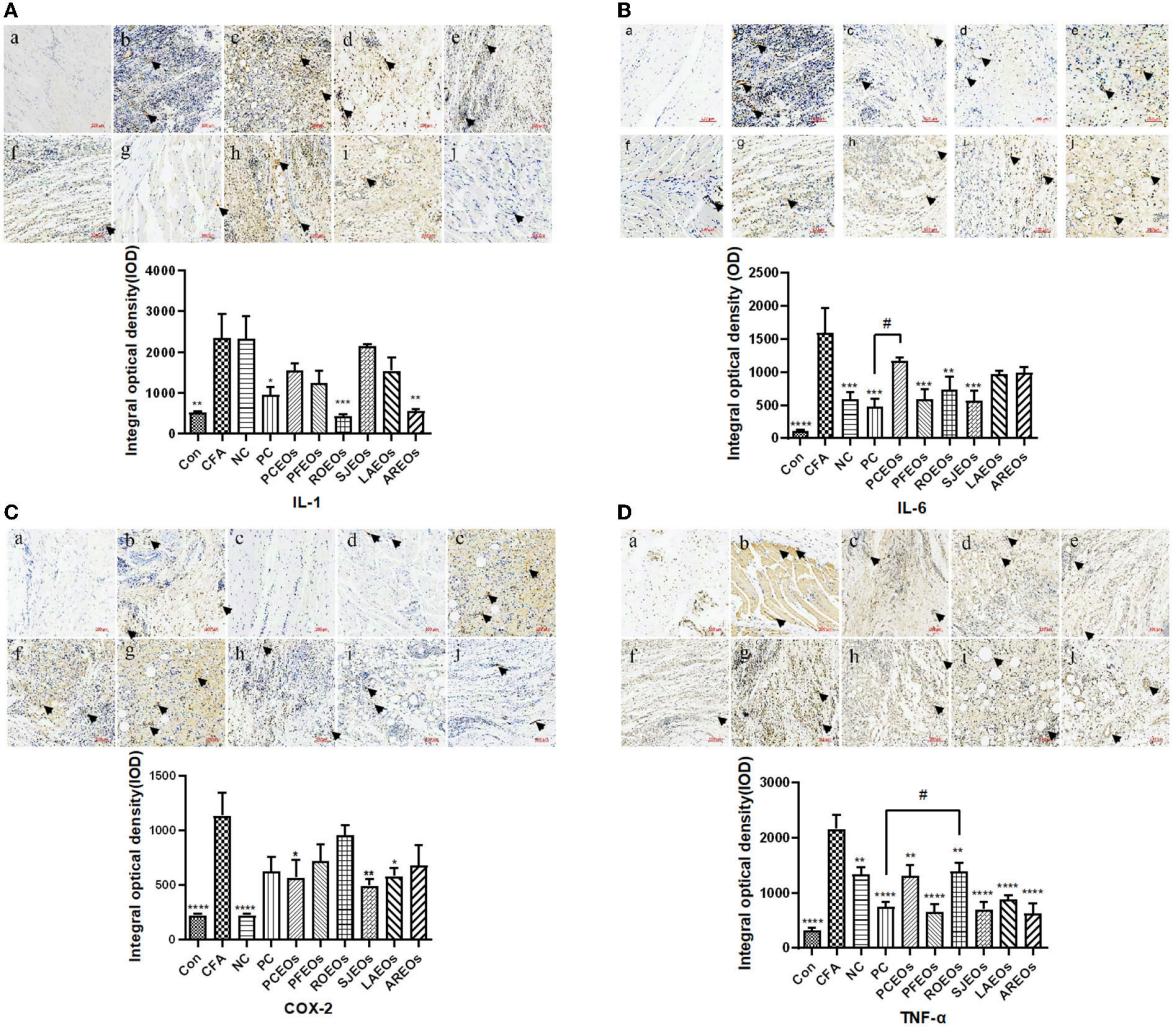
Immunohistochemical staining of rat articular tissues for COX-2 **(A)** IL-1 **(B)**, IL-6 **(C)**, and TNF-α **(D)** (200 × magnification). (a) normal control (Con) group, (b) model (CFA) group, (c) negative control (NC) group, (d) positive control (PC) group, (e) PCEO group, (f) PFEO group, (g) ROEO group, (h) SJEO group, (i) LAEO group, and (j) AREO group. Statistical significance relative to the model (CFA) group is indicated, *p ≤ 0.05, **p ≤ 0.01, ***p ≤ 0.001, ****p ≤ 0.0001, significantly different from the model (CFA) group. Arrows indicate the lesion sites. Statistical significance relative to the positive control (PC) group is indicated, ^#^*p* ≤ 0.05.

Complete Freund's adjuvant can induce numerous inflammatory responses of cytokines, including COX-2, iNOS, IL-1, and IL-6 (Zhang et al., 2020c). To further understand the anti-inflammatory mechanism of these Lamiaceae essential oils, the spatial-temporal expression profiles of inflammatory cytokines in rat articular tissues were investigated (Figure 6). In the model group, CFA treatment greatly induced the expression of COX-2, TNF-α, IL-1, and IL-6 in rat articular tissues, while the essential oil treatments notably reduced that of TNF-α, IL-1, and IL-6 compared to the Con group. Nevertheless, COX-2 expression was slightly decreased after ibuprofen treatment. Caryophyllene was a common component shared by the six essential oils, which was reported to inhibit the expression of TNF, IL-1β, and COX-2 in APP/PS1 mice (Alzheimer-like phenotype) through CB2 receptor activation and the PPARγ pathway (Cheng et al., 2014). In our study, PFEO exhibited the greatest anti-inflammatory capacity inhibiting adjuvant arthritis and largely reduced the expression of inflammatory cytokines including TNF-α, IL-1, and IL-6. Linalool, a dominant component in PFEO, exhibited notably anti-inflammatory potential by reducing the expression of IL-1β and TNF-α in BV2 microglia cell lines (Li et al., 2015). Our results are consistent with those of previous studies, indicating that linalool may be a potential anti-inflammatory compound in those essential oils. In all, our results demonstrated that essential oils of Lamiaceae herbs play a key role in alleviating inflammation by inhibiting inflammatory cytokine expression.

### Antitumor activity of the six essential oils

The antitumor activity was evaluated by examining *in vitro* inhibitory effects of these essential oils on LNCaP and B16 cells, and the results are shown in Table 3. The IC_50_ values of the six essential oils on LNCaP cells were between 116.41 and 189.63 μg/mL; LAEO (116.41 μg/mL) showed the strongest inhibitory effect, followed by AREO (126.20 μg/mL), PFEO (127.90 μg/mL), SJEO (129.40 μg/mL), PCEO (138.24 μg/mL), and ROEO (189.63 μg/mL). The IC_50_ values of these essential oils on B16 cells ranged from 86.91 to 228.91 μg/mL; AREO (86.91 μg/mL) exhibited the highest inhibitory effect, followed by ROEO (93.96 μg/mL), PCEO (109.32 μg/mL), PFEO (144.36 μg/mL), LAEO (152.69 μg/mL), and SJEO (189.63 μg/mL).

**Table 3:** Antitumor activity of six Lamiaceae plants essential oils.

**Essential oil**	**IC**_**50**_ **(**μ**g/ml)** ^**a**^	
	**B16**	**LNCaP**
PCEOs^b^	109.32^ACFc^ ± 9.51 ^d^	138.24^ACDE^ ± 13.74
PFEOs	144.36^BE^ ± 7.75	127.90^ACDE^ ± 18.76
ROEOs	93.96^CF^ ± 8.65	189.63^B^ ± 11.93
SJEOs	228.91^D^ ± 13.89	129.40^CDE^ ± 15.78
LAEOs	152.69^E^ ± 23.23	116.41^DE^ ± 14.50
AREOs	86.91^F^ ± 6.29	126.2^E^ ± 17.79
Hydroquinone	495.5^G^	
Paclitaxel		0.04^F^ ± 0.01

^a^* IC_50_ values of EOs were calculated by MTT assay*.

*^b^ PCEOs, Pogostemon cablin (Blanco) Benth. essential oils, PFEOs, Perilla frutescens (L.) Britton essential oils, SJEOs, Salvia japonica Thunb. essential oils, ROEOs, Rosmarinus officinalis L. essential oils, LAEOs, Lavandula angustifolia Mill. essential oils, AREOs, Agastache rugosa (Fisch. & C. A. Mey.) Kuntze. essential oils.*

*^c^ values followed by different capitals are significantly different (P ≤ 0.05) in One-way ANOVA. ABCDEFG values followed by different capitals are significant different (p ≤ 0.01) in one-way ANOVA.*

*^d^ Data are means ± SD of 3 replications*.

Previous studies have shown that patchouli alcohol, linalool, caryophyllene, borneol, and camphor achieve anticancer effects by inhibiting the expression of inflammatory factors (Santos and Rao, 2000; de Lima et al., 2014; Fidyt et al., 2016; Lian et al., 2018; Oner et al., 2019). Our results showed that the dominant components of six essential oils had different degrees of anticancer effects on B16 and LNCaP cells, consistent with previous studies. PCEO and AREO had better performance in inhibiting B16 and LNCaP cells. Patchouli alcohol, a dominant component of PCEO (43.04%) and AREO (45.70%), maybe a key anticancer ingredient in these two essential oils. Linalool was a dominant component in LAEO (29.84%) and PFEO (66.65%) and may be a key component that plays an important role in their anticancer activity. Borneol (14.59%) and camphor (15.10%) were the major components of ROEO, which were likely the key antitumor component in our *in vitro* cell experiment. Similarly, linalool (8.89%) and camphor (9.54%) may be principal components participating in the anticancer activity of SJEO.

### Antioxidant activity of the six essential oils

**Figure 7:**
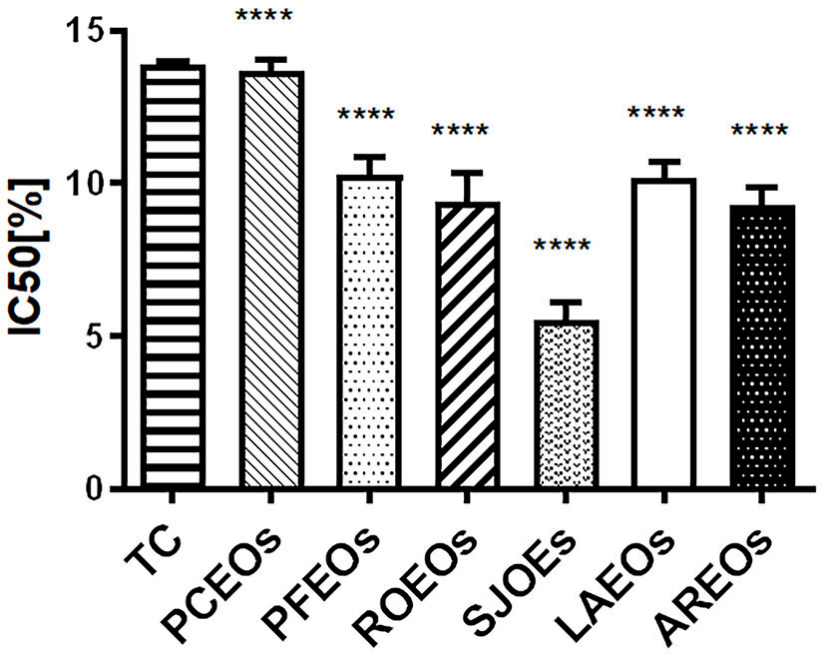
Measurement of DPPH free radical scavenging capacity of the six Lamiaceae essential oils using Trolox C (TC) as the positive control. Statistical significance relative to the Trolox C (TC) group is indicated, ****p ≤ 0.0001, significantly different from the positive control group.

The DPPH method, which is stable, simple, and fast, was employed to assess free radical-scavenging activity of the six essential oils (Luo et al., 2019; Zhang et al., 2020a,b). In this study, our results showed that the IC_50_ values of the six essential oils were between 7.93 and 13.83 μg/mL (Figure 7). SJEO (4.88 μg/mL) showed the highest free radical scavenging capacity, while PCEO had minimal capacity (13.89 μg/mL). The antioxidant activity of AREO (8.79 μg/mL), ROEO (8.98 μg/mL), LAEO (9.80 μg/mL), and PFEO (10.87 μg/mL) was superior to that of Trolox C (13.80 μg/mL).

The antioxidant activity of some Lamiaceae essential oils had been assessed using the DPPH method in the previous study (Spiridon et al., 2011; Luo et al., 2019). Luo et al. (2019) demonstrated that LAEO (IC_50_ = 1.07%) possesses higher antioxidant activity than PCEO (IC_50_ = 1.60%), ROEO (IC_50_, 2.80%), and PFEO (IC_50_, 18.77%). Spiridon et al. (2011) showed that LAEO (IC_50_ = 96.67 μg/mL) can effectively scavenge free radicals. In this study, SJEO (IC_50_ = 4.88 μg/mL) showed the highest activity on scavenging free radicals, followed by AREO, ROEO, LAEO, and PFEO.

The antioxidant activity of essential oils may be largely associated with their major components, among which eucalyptol, α-pinene, and linalool have been reported to demonstrate antioxidant activity (Khan et al., 2014; Oner et al., 2019; Zhang et al., 2020c). For example, α-pinene, with an IC_50_ of 310 μg/mL, was reported to possess antioxidant activity in DPPH assays (Bouzenna et al., 2017). In this study, terpenyl acetate (10.52%), camphor (9.54%), α-pinene (5.97%), linalool (5.97%), and benzyl acetate (5.73%) were the principal components of SJEO, which may account for its high antioxidant activity. Chavibetol (17.72%), camphor (15.10%), verbenone (15.29%), borneol (14.58%), and eucalyptol (6.04%) were the dominant components of ROEO, which may lead to high antioxidant activity. Taken together, our results indicate that some essential oils, as well as their major components, may serve as potent natural antioxidants.

## Conclusion

The demand for essential oils is growing due to their potential applications in pharmacology and industry. The phytochemical composition of essential oils takes charge of their bio-activities and their pharmaceutical effects. However, the chemical composition of plant essential oils is affected by many factors, such as a growing environment and identification methods. In this study, we used modified methods to identify different chemical component profiles of essential oils extracted from six folk medicinal plants. A total of 167 components were identified and analyzed by GC-MS, among which the dominant components were patchouli alcohol, pogostone, linalool, chavibetol, β-caryophyllene, terpenyl acetate, camphor, chavibetol, verbenone, borneol, and γ-terpineol (Table 2). HCA of chemical compositions was first analyzed in six essential oils, which provides more reference for pharmaphylogeny research (Figure 1). Meanwhile, the chemical structure of 15 major compounds was exhibited to provide more reference for later research. Our results were remarkably different from the former reported and provide a new insight into the phytochemical composition of the six essential oils.

The six Lamiaceae essential oils exhibited diverse anti-inflammatory activities on CFA-induced adjuvant arthritis in rats. PFEO, with a high linalool concentration (67.65%), showed higher anti-inflammatory activity relative to the rest of the essential oils and ibuprofen. Anti-inflammation was achieved by inhibiting the expression of COX-2, IL-1, IL-6, and TNF-α. All six essential oils also demonstrated different DPPH radical scavenging capacities except PCEO. SJEO, with high concentrations of terpenyl acetate (10.52%) and camphor (9.54%), showed the highest antioxidant capacity. The six essential oils also exhibited significantly different antitumor activities on LNCaP and B16 cells. AREO, with a high proportion of patchouli alcohol (45.70%), showed the highest antitumor capacity by inhibiting B16 cells with the lowest concentration of 86.91 μg/mL. LAEO, high in linalool (29.84%), showed promising antitumor capacity by inhibiting LNCaP cells at the lowest dosage of 116.5 μg/mL. Collectively, these six Lamiaceae essential oils, possessing varied chemical compositions and biological activities, exhibit potential for serving as bio-functional additives in biomedical products, such as anti-inflammatory and antitumor drugs.

Five of the six medicinal plants belong to the subfamily Nepetoideae (Dumort.) Burnett, except for *P. cablin*, which belongs to the subfamily Lamioideae Harley, indicating that species with similar bio-activities and pharmaceutical effects are clustered in their phylogenetic relationships. More specifically, based on our study, PCEO showed the lowest effects on anti-inflammatory and antioxidant activities, which is consistent with the phylogeny (Supplementary Figure S2). In the views of pharmaphylogeny, species that are closely related are not only similar in physiological characteristics, but at the same time, it is also reflected in the similarity of phytochemical components. Our results showed that the principal components of the six essential oils were greatly distinct. Perhaps because the genes expressing these chemical compositions have suffered different selection pressures during evolution, or they have been of independent origin and have undergone different evolutionary pathways. However, we can still find chemical compositions that match the phylogenetic tree. For example, *L. angustifolia* and *P. frutescens* are sister groups, and the proportion of nerolidol and linalool showed similarity in LAEO and PFEO; *R. officinalis* and *S. japonica* are closely related, and the content of (+)-2-bornanone and eucalyptol showed similarity in SJEO and ROEO.

Although these six Lamiaceae plants are widely used and cultivated in China, only *A. rugosa, P. frutescens* and *S. japonica* are native to China. *L. angustifolia* and *R. officinalis* are native to Mediterranean, and *P. cablin* is distributed around the equator in Southeast Asia. This research provided more references for pharmaphylogeny and drug discovery from folk medicinal plants, and more studies need to be done for further exploring the drugs' function.

## Data availability statement

The original contributions presented in the study are included in the article/Supplementary material, further inquiries can be directed to the corresponding authors.

## Ethics statement

The animal study was reviewed and approved by the animal experiments were carried out following Ethical Guidelines of the Laboratory Animal Center of Sun Yat-sen University.

## Author contributions

YK: data curation, writing–original draft, and writing–review and editing. LG: conceptualization and supervision. JS: data curation and software. PS: software and visualization. CK: data curation. LZ: conceptualization and methodology. All authors contributed to the article and approved the submitted version.

## Funding

This study was supported by the National Key Research and Development Program of China (No. 2017YFC1700701), CACMS Innovation Fund (CI2021A03909), and the Key Laboratory of Biology and Genetic Improvement of Horticultural Crops, the Ministry of Agriculture and Rural Affairs, China.

## Conflict of interest

The authors declare that the research was conducted in the absence of any commercial or financial relationships that could be construed as a potential conflict of interest.

## Publisher's note

All claims expressed in this article are solely those of the authors and do not necessarily represent those of their affiliated organizations, or those of the publisher, the editors and the reviewers. Any product that may be evaluated in this article, or claim that may be made by its manufacturer, is not guaranteed or endorsed by the publisher.

## Abbreviations

AREO: the essential oil of *Agastache rugosa* (Fisch. & C. A. Mey.) Kuntze
LAEO: the essential oil of *Lavandula angustifolia* Mill.
PCEO: the essential oil of *Pogostemon cablin* (Blanco) Benth.
PFEO: the essential oil of *Perilla frutescens* (L.) Britton
ROEO: the essential oil of *Rosmarinus officinalis* L.
SJEO: the essential oil of *Salvia japonica* Thunb.
