# Supplementary material for: Chemical composition and biological activities of essential oils from six lamiaceae folk medicinal plants
Source: Front Plant Sci. 2022 Aug 1;13:919294. doi: 10.3389/fpls.2022.919294 (PMC9376358; doi:10.3389/fpls.2022.919294)
Supplement: Supplementary file 1 [file Data_Sheet_1.PDF]

Supplementary materials:

The TIC figures of six essential oils detected by GC-MS.

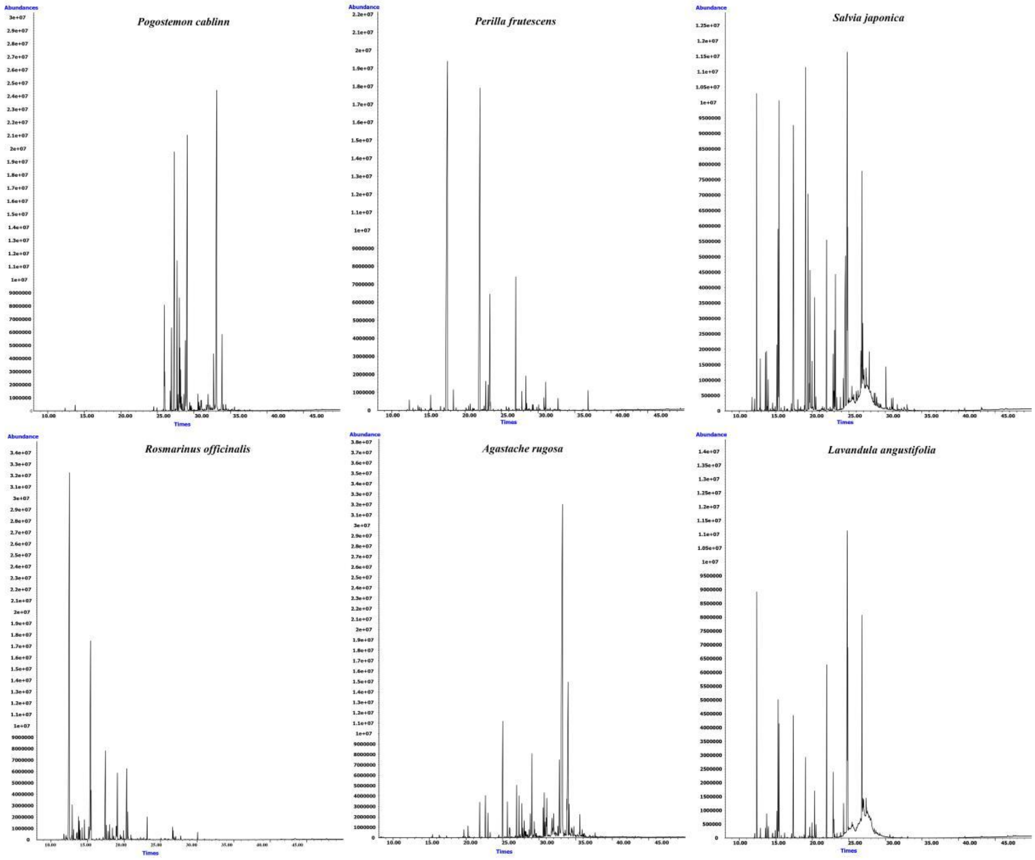

Figures S1 The TIC figures of six essential oils detected by GC-MS.

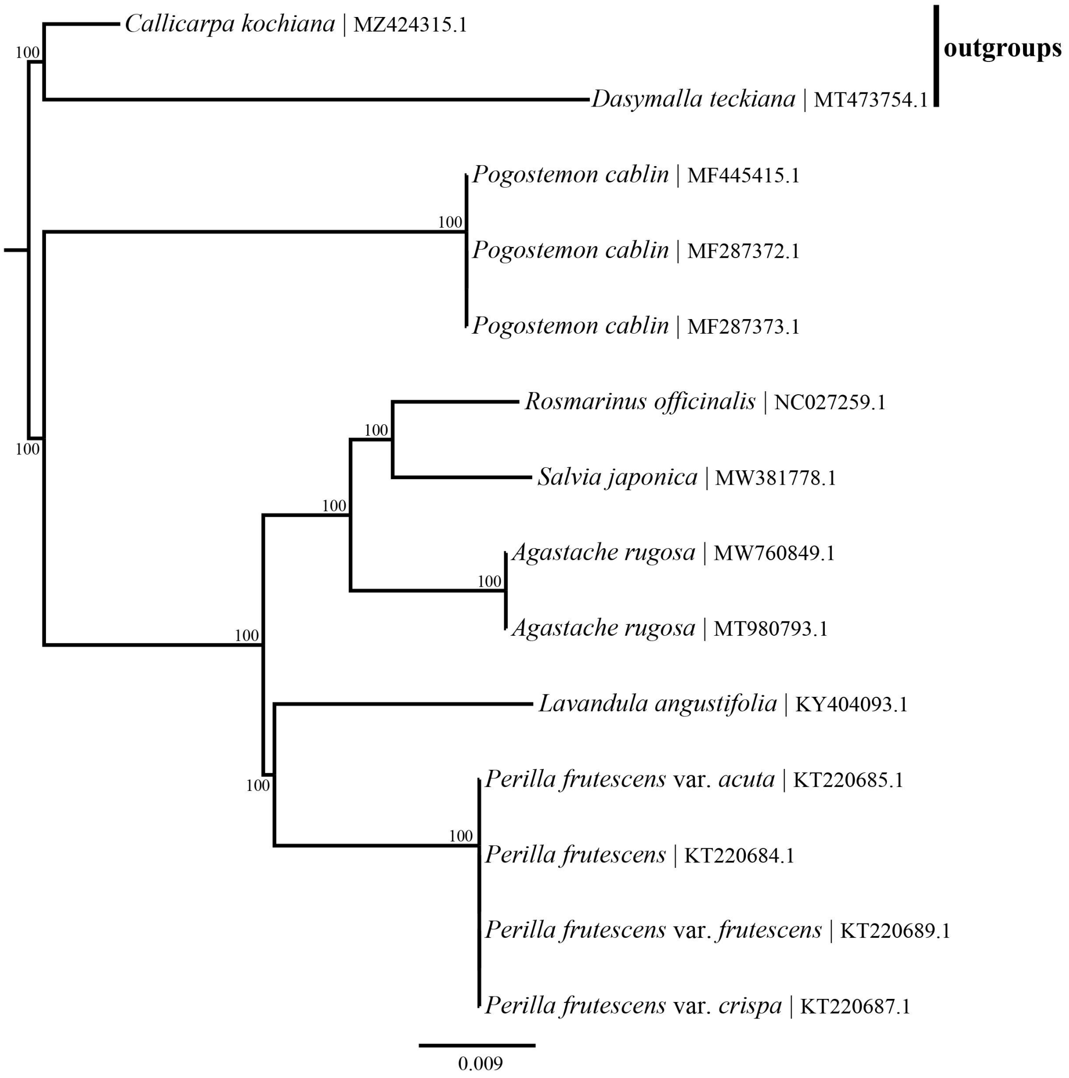

Figure S2. Phylogenetic tree of six Lamiaceae medicinal plants based on Chloroplast genomes. ML bootstrap support values are shown at each node.
